# Effects of conditioning on the left ventricular function of young purebred Arabian horses

**DOI:** 10.1371/journal.pone.0304724

**Published:** 2024-06-03

**Authors:** Gabriel Vieira Ramos, Maíra Moreira Santos, Fábio Nelson Gava, José Corrêa de Lacerda-Neto

**Affiliations:** 1 Department of Veterinary Clinics and Surgery, School of Agricultural and Veterinary Sciences, São Paulo State University (UNESP), Jaboticabal, São Paulo, Brazil; 2 Department of Veterinary Clinics, State University of Londrina, Londrina, Paraná, Brazil; SDM College of Medical Sciences and Hospital, Shree Dharmasthala Manjunatheshwara SDM University, INDIA

## Abstract

The effects of conditioning on cardiac function in young horses is still unknown. For this reason, this study evaluated the left ventricular (LV) function of young horses by echocardiography after six weeks of conditioning. Fourteen untrained young purebred Arabian horses were evaluated at rest and after a stress test (ST) before and after a six-week conditioning program. There was an increase in V_4_ (p < 0.001) after conditioning, as well as a reduction in both heart rate (HR) at rest and peak HR during the ST (p < 0.001). There was also a reduction in internal diameter, along with an increase in interventricular septal, free wall and mean thicknesses and LV mass (p < 0.05). After the ST, the conditioned animals showed higher values of velocity time integral, stroke volume, systolic and cardiac indices, ejection (ET) and deceleration times (DT), end-diastolic volume, time to onset of radial myocardial velocity during early diastole and time to peak of transmitral flow velocity, in addition to reduced pre-ejection period (PEP), PEP/ET ratio and mean velocity of circumferential fiber shortening (p < 0.05). The conditioning protocol promoted physiological adaptations that indicate an improvement in the animals’ aerobic capacity associated with an enhanced left ventricular function.

## Introduction

It is reported that physical exercise causes a modification in homeostasis, mainly due to the increase in metabolic demand, leading to adaptive responses in a wide variety of organs, systems and physiological processes, as well as conditioning when induced in a structured and constant way [1]. More specifically in the heart, physical conditioning can promote structural and functional changes. Morganroth (1975) [2] was the first to characterize different patterns of cardiac remodeling in human athletes according to the nature of the sport. As reported, there are two types of remodeling: eccentric and concentric. The first, whose main characteristic is the enlargement of the ventricular chambers with small changes in the intraventricular septal and free wall thicknesses, is mainly observed in athletes who practice isotonic exercises (i.e., marathon runners, cyclists, rowers, etc.) and is related to significant increases in preload. The second, on the other hand, is characterized by high values of free wall and interventricular septal thicknesses associated with a reduction in the ventricular chambers and is mainly observed in athletes who practice isometric exercises (i.e., weightlifters, bodybuilders, etc.), in which there are significant increases in afterload [2–6].

Similarly, conditioning promotes several morphofunctional adaptations in horses, leading to an enhanced maximum O_2_ consumption ($\dot{\text{V}}{\text{O}}_{2}\text{max}$) [1]. For this reason, several training protocols are described based on the determination of some physiological variables, such as $\dot{\text{V}}{\text{O}}_{2}\text{max}$ [7], percentage of maximum heart rate (%HRmax) [8] and plasma lactate concentration [9], through an incremental exercise test (IET). With respect to lactate, the tracing established by the lactate concentrations determined at higher speeds reflects the predominant metabolic pattern in each interval that precedes the determination [10].

The IET performed on a treadmill takes into account the gradual increase in external load through incremental increases in speed associated or not with an incline. As the IET progresses, there is enhancement of energy demands and the metabolic participation of anaerobic pathways, which results in the exponential increase in lactate concentration from a certain point [11]. This inflection point, known as lactate threshold (LT), has a corresponding speed that is representative of the maximum exercise intensity at which there is a balance between lactate production and removal from the blood [12]. The exercise performed under load corresponding to LT leads to plasmatic lactate concentrations that are considered constant (Δ[lactate] < 1.0 mmol/L). This value is called maximal lactate steady state (MLSS). The confirmation of MLSS requires tests that last over 30 minutes at the LT speed, repeated at least three times with small adjustments in speed when necessary–factors that limit the use of such a parameter from a practical point of view. For this purpose, the so-called fixed methods, which are performed in a single session, are commonly used to estimate the MLSS, including the speed at which the lactate concentration reaches 2 mmol/l (V_2_). This method is considered a good predictor of MLSS in horses, whether in the field [13] or on a treadmill [14], and can be used to establish and evaluate conditioning programs.

Regarding the cardiovascular system, in athletic horses the eccentric remodeling is mainly reported for different modalities and breeds [15–17]. As a consequence, the structural modifications promoted by exercise alter their cardiac function and characterize a condition called "athlete’s heart" [18]. These modifications, when evaluated at rest, may present echocardiographic characteristics similar to certain heart diseases [19]. In this context, echocardiography can be associated with physical activity as an auxiliary method to differentiate heart diseases from adaptations promoted by training, which cannot be detected in the echocardiographic examination at rest [20, 21]. Such association is known as stress echocardiography, which, in addition to exercise, can be performed during or after pharmacological protocols in order to cause positive chronotropic and inotropic effects on the heart, allowing its assessment under stress. In horses, it is recommended that cardiac examination after stress tests (physical or pharmacological) be performed within 120 seconds and at a heart rate (HR) above 100 bpm [21]. As an advantage, stress echocardiography is a simple and physiological method with high sensitivity, accuracy and specificity [22]. Since 2005, equine medicine has incorporated stress echocardiography as a complementary method to cardiovascular evaluation, a procedure widely used in human medicine [23]. Among the various methods described for echocardiographic evaluation both at rest and after stress, the B-mode, M-mode and Doppler principle [24–26], such as pulsed-wave (PW) Doppler and tissue Doppler imaging (TDI) [26]. Furthermore, the echocardiogram can be applied to assess the left ventricle (LV) systolic and diastolic functions. In horses, other echocardiographic methods have been used, e.g., TDI to assess the LV radial wall motion [27, 28], complementing PW-Doppler assessment [29–31].

Thus, the present study aims to evaluate cardiac adaptations in young purebred Arabian (PBA) horses by transthoracic echocardiography performed at rest and immediately after physical stress. The animals, which had no history of supervised exercise and conditioning, were submitted to a V_2_-based treadmill training protocol for six weeks. We hypothesize that the conditioning program will promote an increase in the horses’ aerobic capacity associated with cardiac adaptations compatible with the so-called "athlete’s heart", along with improved LV systolic and diastolic functions both at rest and immediately after physical exercise.

## Materials and methods

### Animals

A total of 14 purebred Arabian horses aged (mean ± SD) 28.42 ± 3.75 months with a body mass (BM) of 313.3 ± 17 kg and a thoracic circumference (TC) of 154.9 ± 4 cm were used. The animals belonged to private breeders and consent forms from animal owners were obtained prior to the studies. The present research study was approved by the Ethics Committee on the Use of Animals of the School of Agricultural and Veterinary Sciences (CEUA–FCAV) under protocol no. 1795/21. The horses that were tested negative for equine infectious anaemia and glanders were used in the study. The inclusion criteria were: purebred Arabian horses, healthy, aged less than 36 months, with no previous history of forced physical exercise or participation in a conditioning program, and without participation in any drug treatment. Prior to the beginning of the experimental period, all animals had their health condition verified through a complete physical examination, blood count and cardiovascular evaluation by electrocardiography and echocardiography at rest. The horses were kept on a 100 x 70 m Tifton grass paddock, where they were offered coast cross hay and commercial concentrate containing 60% of the energy requirement for moderate exercise (approximately 11.13 Mcal/animal/day) [32], as well as water *ad libitum*.

Before the beginning of the experimental period, the animals were acclimatized to the treadmill for five days, when they were individually led to walk at their own pace on the 5-meter rolling belt with the treadmill turned off. Afterwards, the 14 horses walked on the treadmill in line, going around the building three times. On the following day, they were adapted to the girth and required to walk at their own pace again, so that all passed over the rolling belt with the treadmill still turned off, also going around the building three times. On the third day, with the girth attached and secured to the treadmill safety belt, the horses were placed on the treadmill, which was then turned on at a speed that allowed them to walk at their own pace for 15 minutes. On the fourth day, the previous procedure was repeated, but at a higher speed to encourage trotting. On the fifth and last day of adaptation, the horses were induced to perform a canter both in the horizontal plane and at a 2% incline.

### Incremental Exercise Test (IET)

Two IETs were applied, before and after the six-week conditioning period, to deter-mine the values of V_2_ and V_4_ (defined as the external load necessary for the plasma lactate concentration to reach 2 and 4 mmol/L, respectively). During each IET, electrocardiograms were recorded using a digital electrocardiograph with 12 leads at a sampling frequency of 1200 Hz (ECGPC Vet, Tecnologia Eletrônica Brasileira, São Paulo, Brazil), of which only the frontal leads (DI, DII, DIII, aVR, aVL and aVF) were stored. Each IET preceded a 10-minute warm-up period subdivided into two 5-minute steps at 1.7 m/s and 3.5 m/s, respectively, without any incline. After the warm-up, the treadmill was inclined at 3% (~ 1.84°) at a speed of 5 m/s, and after each 5-minute step the speed was increased by 0.5 m/s. Between the steps, the treadmill was turned off for 1 minute to collect blood samples. When the blood lactate concentration reached values equal to or greater than 4 mmol/L, the test was concluded and immediately followed by the cooling-down period for 10 minutes, which was subdivided into two steps of 5 minutes at 3.5 m/s and 1.7 m/s, respectively, without any incline.

### Stress Test (ST)

Two stress tests (STs), before and after the six-week conditioning period (both after each IET), were applied. The intervals between the IETs and their respective STs were ap-proximately 72 hours. The protocol used was similar to that proposed by Lorello *et al*. (2019) [33], with adaptations for treadmill. The STs were performed in the horizontal plane, starting with a previous warm-up period of 10 minutes at 1.7 m/s and another 10 minutes at 3.2 m/s and then proceeding with 30 minutes of exercise at 5.8 m/s. Subsequently, the first sprint was performed for 3 minutes at 7.5 m/s, followed by 5 minutes of trotting at a speed between 3.0 and 3.5 m/s (adjusted according to the animal’s comfort during walking). Next, the animals galloped again for 2.5 minutes at a speed of 8.5 m/s (second sprint). At the end of the test, the treadmill was turned off and the echocardiographic examination was immediately performed. Finally, the cooling-down was carried out at 1.7 m/s for 10 minutes. The HR was recorded during the echocardiographic examination, with concomitant single-lead ECG recording.

The echocardiographic examination was initiated in the left parasternal window (LPW), followed by images of the right parasternal window (RPW). The times needed to completely stop the treadmill (T1), obtain images in the LPW (T2), move the ultrasound device (T3) and obtain images in the RPW (T4) were recorded. The echocardiographic examination was performed in the following sequence: 1) use of PW-Doppler on longitudinal images obtained through the LPW and 2) evaluations in B- and M- modes and TDI on longitudinal and cross-sectional images obtained through the RPW. The ST protocol is summarized in S1 Table.

### Blood sample collection and processing

All blood samples analyzed were obtained during the IETs. Prior to each IET, the animals were submitted to right jugular venocatheterization using an aseptically implanted sterile 14G x 20 cm polyurethane catheter (U/L subclavian CVC, Biomedical, São Paulo, Brazil), to which a 120 cm extension tube (Medsonda, Paraná, Brazil) filled with physiological solution for patency maintenance was attached. Before each collection, the system was cleaned by aspiration of 20 ml of content. Afterwards, 2 ml of blood was collected and immediately transferred to tubes (BD Vacutainer, BD, New Jersey, USA) containing sodium fluoride/Na_2_EDTA (3mg/6mg). At the end of each collection, the system was filled again with 20 ml of saline solution. During the IET, part of the total blood samples was subjected to lactate concentration measurement on an electro-enzymatic bioanalyzer (YSI 2300 Lactate Analyzer, Yellow Springs Instruments, Ohio, USA) prepared with cell lysing agent (YSI 1515 Cell Lysing Agent, Yellow Springs Instruments, Ohio, USA). The rest of the samples was centrifuged at 3000 rpm (1500 g) for 10 minutes for plasma suspension. The plasma samples were frozen at -15 °C, and, within 24 hours, were subjected to lactate concentration measurement by the same method previously described, but without the addition of cell lysing agent. The lactate concentrations measured in blood samples were used only as a parameter for conducting the IET. The plasma lactate concentrations were used in the statistical analysis and will be later discussed.

### Treadmill conditioning

The adopted conditioning program lasted six weeks, during which each animal per-formed, on alternate days, five biweekly sessions of 40 minutes, totaling 15 sessions. The training speed, individually determined in the first IET, was based on V_2_. In order to minimize the occurrence of injuries, the conditioning program was implemented gradually, as follows: 1st to 3rd session at a speed of 80% of the V_2_ in the horizontal plane; 4th to 6th session at a speed of 80% of the V_2_ and an incline of 3%; 7th to 9th session at a speed of 100% of the V_2_ and an incline of 3%; and between the 10th and the 15th session at a speed of 100% of the V_2_ and an incline of 5% (~ 2.76°). Each training session consisted of a 10-minute warm-up period, subdivided into two steps of 5 minutes at 1.6 m/s and 3.4 m/s, respectively, without any incline. The training period consisted of 40 minutes of exercises, subdivided into three 12-minute steps at a speed and incline established in each the session, intercalated by two 2-minute steps for pace change by in-creasing or decreasing the speed without any incline. Such steps were proposed to mitigate repetitive effort and consequent injuries. At the end of the training period, the cool-down was performed at 1.6 m/s for 5 minutes without any incline. The detailed conditioning protocol can be seen in S2 Table.

### Echocardiography

The horses were submitted to transthoracic echocardiography on the right and left sides using an ultrasound system (MyLabAlpha, Esaote SpA, Genoa, Italy) and a 1 to 4 MHz multi-frequency sector transducer (SP2430, Esaote SpA, Genoa, Italy) with a maxi-mum penetration of 35 cm. The evaluation was carried out before and after the conditioning period in two situations, namely: 1) at rest prior to the ST, when the animals were kept in the Equine Sports Medicine Laboratory in stocks on a rubber floor, in standing position, without the use of tranquilizers; and 2) immediately after the second run and still on the treadmill, which remained turned on. Measurements were per-formed in B-mode using PW-Doppler and TDI, as well as in M-mode guided by B-mode. For each variable, the measurements were carried out in three consecutive cardiac cycles. All variables analyzed and calculations used are described in S3 Table.

#### B-mode

B-mode measurements were performed from longitudinal images obtained in the RPW according to techniques previously described for horses [31, 34]. Before the examination, the middle regions between the shoulder level and the elbow, both left and right, were shaved and cleaned with subsequent application of contact gel (Carbogel ULT, Carbogel, São Paulo, Brazil) for ultrasonic transmission. The transducer was positioned perpendicular to the animal’s thorax and over the right fourth intercostal space (ICS) in order to direct the ultrasound beam to the left fourth ICS, with slight clockwise rotation. The image formed, called longitudinal 5-chamber (L5C), allowed the visualization of the following structures: right atrium (RA), tricuspid valve (TV), right ventricle (RV), interventricular septum (IVS), left atrium (LA), mitral valve (MV), left ventricle (LV), LV outflow tract (LVOT), aortic valve (AV) and aorta (Ao). In this image, the aortic diameter was measured in the aortic annulus region during peak ventricular systole–the moment within the cardiac cycle when the smallest diameter of the left ventricular chamber is observed, with the AV completely open [35]. Subsequently, the ultrasound beam was directed caudally to-wards the fifth left ICS. The image formed, known as longitudinal 4-chamber (L4C), was comprised of the RA, TV, RV, IVS, LA, MV and LV. In the L4C image, the LA diameter was measured at the end of ventricular systole, when the LV internal diameter (LVID) was the smallest within the cardiac cycle or one frame before the VM opening. All measurements were made using the leading-edge-to-leading edge method.

#### M-mode

M-mode measurements were made from a right parasternal short-axis image at the chordal level. The end-systolic (s) and diastolic thicknesses of IVS (IVSs and IVSd), LVID (LVIDs and LVIDd) and LV free wall (LVFWs and LVFWd) were evaluated. After determination of the linear values, the LV fractional shortening (FS) was calculated using the following equation [36]:

$$\text{FS}\left(\text{\%}\right)=\left(\text{LVIDd}-\text{LVIDs}\right)/\text{LVIDd})\text{x}100$$ (1)

The LV end-systolic and diastolic volumes (ESV and EDV, respectively) were also estimated by applying the equation modified by Teichholz *et al*. (1976) [37], which proved to be adequate for horses [38, 39]:

$$\text{V}\left(\text{cm}³\right)=\left(7\text{x}\left(\text{LVID}\right)³\right)/\left(2.4+\text{LVID}\right)$$ (2)

After determination of the ESV and EDV values, the LV ejection fraction (EF) was calculated according to the following equation:

$$\text{EF}\left(\text{\%}\right)=\left(\left(\text{Vd}-\text{Vs}\right)/\text{Vd}\right)\text{x}100$$ (3)

The mean (MWT) and relative (RWT) left ventricular free wall thicknesses and the LV mass were also calculated [17, 40]:

$$\text{MWT}=\left(\text{LVFWd}+\text{IVSd}\right)/2$$ (4)

$$\text{RWT}=\left(\text{LVFWd}+\text{IVSd}\right)/\text{LVFWd}$$ (5)

$$\text{LVmass}=1.04\text{x}\left(\left[\text{LVIDd}+\text{LVFWd}+\text{IVSd}\right]³-\text{LVIDd}³\;\right)–13.6$$ (6)

### Use of Doppler

#### Pulsed-wave Doppler

The measurements carried out included variables related to the LV systolic and diastolic functions and were performed from longitudinal images obtained in the LPW using PW-Doppler. To record the aortic flow, a sample volume of 2 mm was positioned in the AV opening region. From the spectrum, it was possible to assess the following variables: velocity-time integral (VTI) of aortic flow, stroke volume (SV), systolic index (SI), cardiac output (CO), cardiac index (CI), maximal velocity of aortic flow (Vmax), time between on-set and peak aortic flow (TPP), deceleration time (DT) of the aortic flow, time between on-set of QRS complex and beginning of LV ejection phase (pre-ejection period; PEP), LV ejection time (ET), PEP/ET ratio and mean velocity of circumferential fiber shortening (Vcf) [41].

To determine the LV diastolic function also from the longitudinal image obtained in the LPW over the fourth ICS, the sample volume was positioned in the VM opening region. Thus, the LV filling pattern was assessed by analyzing the E waves (rapid filling phase) and A waves (due to LA systole) and calculating the E/A ratio in the same cardiac cycle.

#### Tissue Doppler imaging

To perform TDI, an LV transverse image obtained in the RPW at the chordal level was acquired. A sample volume of 2 mm was positioned in the LVFW between the papillary muscles to cover the subendocardial region during diastole and remain in the myocardium during systole. The evaluation of the LV systolic function consisted of determining the following variables: maximum radial myocardial velocity during isovolumic contraction (S_1_) and the ejection phase (S_m_), time to peak velocity of S1 (*t*S_1_), time to peak velocity of S_m_ (*t*S_m_) and isovolumic contraction time (IVCT).

The LV diastolic function was studied by analyzing the variables maximal isovolumic relaxation (E_1_), early-diastolic radial wall motion (E_m_) and late-diastolic radial wall motion velocities (A_m_), E_m_/A_m_ ratio, E/E_m_ ratio, deceleration time of early-diastolic velocity (DTE_m_), isovolumic relaxation time (IVRT) and time between onset of QRS complex and beginning of early-diastolic velocity (*t*Em) [28]. Based on the IVCT and IVRT values, the myocardial performance index (MPI) was calculated according to the following equation [42]:

$$\text{MPI}=\left(\text{IVCT}+\text{IVRT}\right)/\text{ET}$$ (7)

### Statistical analysis

The values of V_2_ and V_4_ were determined by exponential regression, considering plasma lactate concentration as the dependent variable and speed as the independent variable. Regarding the echocardiographic analyses, for each variable obtained (at rest and immediately after the ST), three measurements were performed in consecutive cardiac cycles, followed by calculation of their mean values. The means for IVSd, IVSs, LVIDd, LVIDs, LVFWd and LVFWs were indexed to BW and TC, according to the allometric equation proposed by Cornell *et al*. (2004) [43]:

$$\text{a}=\text{Y}/{\text{M}}^{\text{b}},$$ (8)

where Y is the measured value and M is the animal’s BW or TC value raised to the power of b. The resulting a value represents the measured variable indexed (or normalized) to BW or TC. For PBA horses, the a and b values were previously calculated [44]. The normality of the residuals was evaluated by visual inspection of the histogram and the Shapiro–Wilk test, while the homogeneity of the variances was analyzed by the Bartlett’s test. Variables considered parametric had their means compared using the Student’s t-test, whilst non-parametric data were compared using the Wilcoxon signed rank sum test, both adjusted for paired samples. The values obtained at rest and after physical stress were not compared to each other, but individually before and after the conditioning program. To carry out all analyses and plot the graphs, RStudio software (2023.03.1+446 "Cherry Blossom" for Windows, Posit Software, Boston, EUA) was used. The significance level was established at 5%.

## Results

Two horses were excluded from the study due to injuries that occurred in the region of the metacarpophalangeal joint of the left forelimb. When detected, the animals were promptly referred to the Veterinary Hospital “Governador Laudo Natel” for clinical evaluation. Both remained at rest for the appropriate drug treatment, and therefore discontinued the experimental protocol.

**Fig 1:**
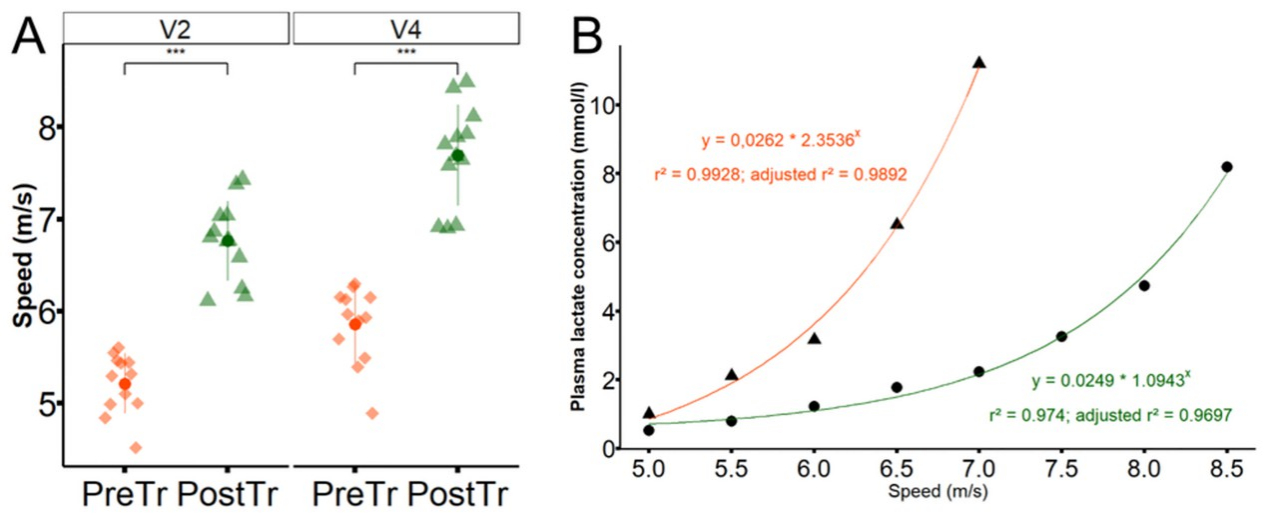
Modification of V2 and V4 after a six week conditioning program. (A) Mean ± SD (pointrange) of the speeds nedeed to reach plasma lactate concentrations of 2 (V_2_) and 4 (V_4_) mmol/l before (PreTr) and after (PostTr) the treadmill conditioning period. *** p < 0.001. (B) Values of plasma lactate concentrations of a subject obtained during the incremental tests per-formed before (triangles) and after conditioning (circles) and their respective exponential curves (lines and equations in orange and green, respectively; p < 0.05).

**Fig 2:**
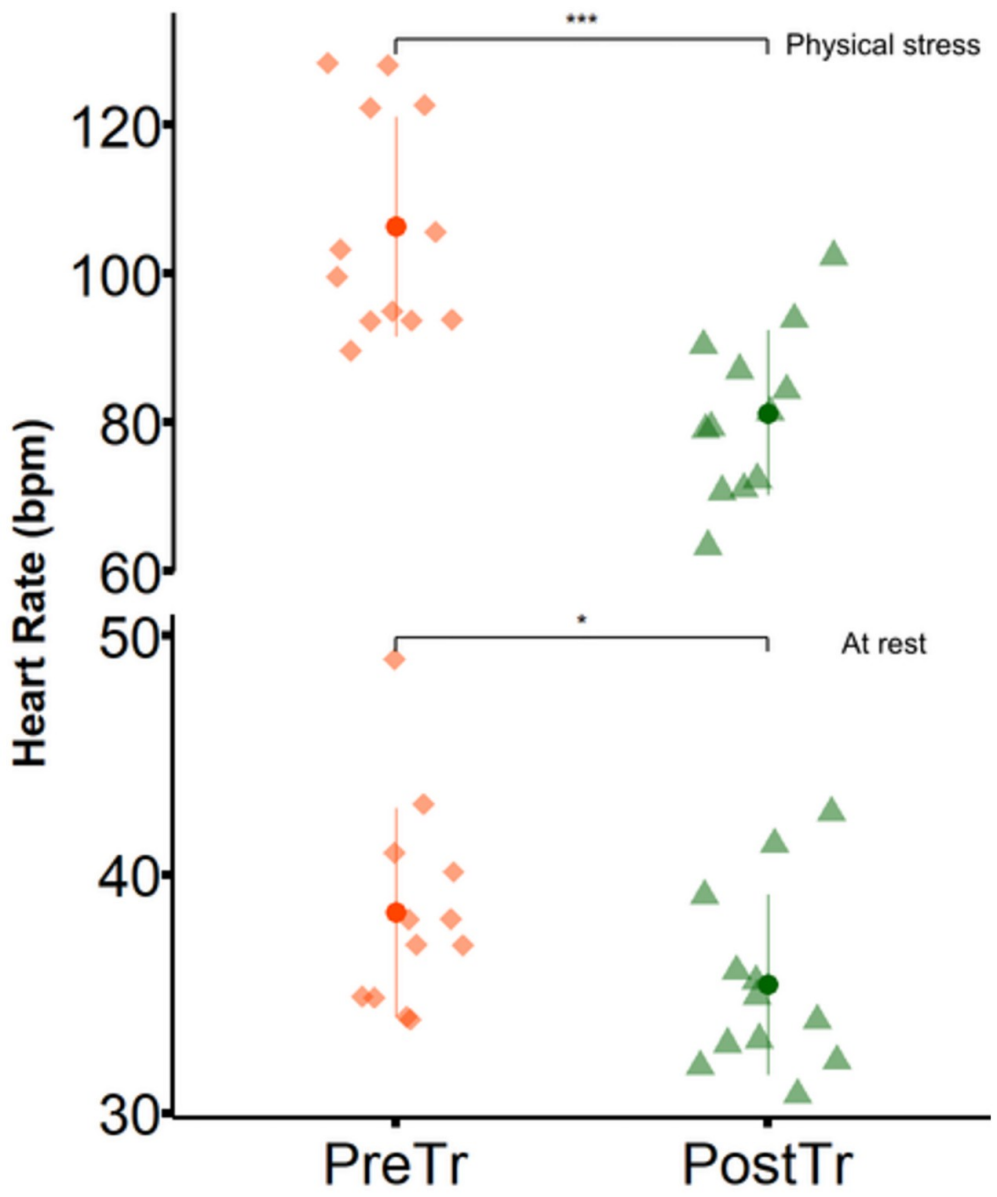
Effects of conditioning on heart rate at reste and immediately after physical stress. Mean ± SD (pointrange) of heart rate values obtained at rest (lower) and immediately after treadmill stress test (upper) before (PreTr) and after conditioning (PostTr). * p < 0.05; *** p < 0.001.

The conditioning protocol used promoted physiological adaptations in the animals, as seen in Fig 1A. There was an increase in the mean values of V_2_ (p < 0.001) and V4 (p < 0.001) obtained in the IET performed after conditioning. Fig 1B shows a shift to the right of the lactate-velocity curve of a subject obtained by exponential regression (p < 0.05). A significant increase in BW (313.3 ± 17 kg vs. 321.2 ± 20 kg; p = 0.008) and TC (155 ± 4 cm vs. 158 ± 5 cm; p = 0.004) was observed during the experimental period. The mean values of HR, on the other hand, reduced after conditioning, both at rest (p < 0.05) and immediately after exercise (p < 0.001), as illustrated in Fig 2.

The influence of conditioning training on the increase in HR promoted by ST was also analyzed. In non-conditioned animals, the peak HR reached in the ST was 102 ± 20 bpm, with a variation of 64 bpm in relation to the rest HR (38 ± 4 bpm; p < 0.001). After conditioning, the enhancement of HR was also significant, with a variation of 43 bpm (p < 0.05) between the rest HR (35 ± 4 bpm) and immediately after the ST (78 ± 12 bpm).

The linear measurements obtained by echocardiographic examination without indexing and indexed to both BW and TC are shown in Table 1. Due to the use of the equation, the indexed values are presented without measurement units.

**Table 1:** Mean ± sd absolute and BW- and TC-indexed linear measurements of young purebred Arabian horses obtained at rest and immediately after physical stress in the pre- (PreTr) and post-conditioning (PostTr) periods.

AT REST									
	Mean ± sd (cm)			Mean ± sd (indexed to BW)			Mean ± sd (indexed to TC)		
	PreTr	PostTr	p	PreTr	PostTr	p	PreTr	PostTr	p
Ao	4.31 ± 0.379	4.477 ± 0.405	0.351						
LA	8.813 ± 0.935	9.045 ± 0.676	0.332						
IVSd	2.513 ± 0.248	2.580 ± 0.227	**0.031**	0.318 ± 0.033	0.323 ± 0.029	0.224	0.013 ± 0.002	0.013 ± 0.001	0.912
LVIDd	9.508 ± 0.568	9.357 ± 0.410	0.224	1.274 ± 0.071	1.242 ± 0.063	**0.041**	0.05 ± 0.003	0.048 ± 0.003	**0.014**
LVFWd	1.883 ± 0.272	2.164 ± 0.175	**0.012**	0.142 ± 0.018	0.161 ± 0.013	**0.023**	0.003 ± 0.001	0.003 ± 0.001	0.234
IVSs	3.701 ± 0.212	3.981 ± 0.219	**0.002**	0.264 ± 0.014	0.280 ± 0.014	**0.012**	0.013 ± 0.001	0.014 ± 0.001	**0.043**
LVIDs	5.998 ± 0.612	5.789 ± 0.637	0.331	0.901 ± 0.089	0.863 ± 0.101	0.233	0.068 ± 0.007	0.064 ± 0.008	0.164
LVFWs	2.939 ± 0.512	3.339 ± 0.375	**0.013**	0.279 ± 0.049	0.313 ± 0.031	**0.022**	0.0051 ± 0.001	0.0052 ± 0.001	**0.041**
MWT	2.199 ± 0.171	2.372 ± 0.129	**0.004**						
RWT	0.473 ± 0.064	0.508 ± 0.037	0.065						
LVmass (g)	1891 ± 234	2052 ± 186	**0.034**						
AFTER PHYSICAL STRESS									
	Mean ± sd (cm)			Mean ± sd (indexed to BW)			Mean ± sd (indexed to TC)		
	PreTr	PostTr	p	PreTr	PostTr	p	PreTr	PostTr	p
Ao	4.406 ± 0.622	4.560 ± 0.228	*0*.*913*						
LA	8.293 ± 0.792	8.837 ± 0.764	0.082						
IVSd	2.376 ± 0.244	2.583 ± 0.197	**0.049**	0.3 ± 0.033	0.323 ± 0.021	0.073	0.013 ± 0.001	0.013 ± 0.001	0.132
LVIDd	9.125 ± 0.362	9.6 ± 0.606	**0.049**	1.236 ± 0.047	1.274 ± 0.084	0.271	0.048 ± 0.002	0.05 ± 0.003	0.244
LVFWd	1.973 ± 0.239	2.095 ± 0.231	0.223	0.149 ± 0.016	0.156 ± 0.017	0.273	0.003 ± 0.001	0.003 ± 0.001	0.733
IVSs	3.698 ± 0.234	4.149 ± 0.25	**< 0.001**	0.263 ± 0.018	0.292 ± 0.018	**0.001**	0.013 ± 0.001	0.014 ± 0.001	**0.006**
LVIDs	5.868 ± 0.282	6.084 ± 0.548	*0*.*072*	0.881 ± 0.049	0.907 ± 0.085	0.334	0.066 ± 0.004	0.067 ± 0.006	0.543
LVFWs	2.998 ± 0.336	3.264 ± 0.365	0.083	0.283 ± 0.027	0.306 ± 0.034	0.131	0.005 ± 0.001	0.005 ± 0.001	*0*.*831*
MWT	2.150 ± 0.116	2.339 ± 0.123	**0.005**						
RWT	0.476 ± 0.027	0.490 ± 0.051	0.408						
LVmass (g)	1718 ± 185	2094 ± 187	**0.001**						

LA: left atrium diameter; Ao: aortic diameter; LVIDd: left ventricle (LV) internal diameter at the end of diastole; LVIDs: LV internal diameter at the end of systole; MWT: mean wall thickness; RWT: relative wall thickness; LVmass: left ventricle mass; LVFWd: LV free wall thickness at the end of diastole; LVFWs: LV free wall thickness at the end of systole; IVSd: intraventricular septal thickness at the end of diastole; IVSs: intraventricular septal thickness at the end of systole. P-values and p-values in italics were obtained by Student’s t-test and Wilcoxon signed-rank test, respectively. P-values in bold indicate a statistical significance of 5%.

The systolic and diastolic variables obtained at rest can be found in Table 2. With regard to the systolic function, only S_m_ showed a statistical difference after the training period, while for the diastolic function there were changes in E_1_, E_m_, A_m_, E, and E/A ratio. The other variables did not show significant differences.

**Table 2:** Mean ± SD and first and third quartiles of the variables of left ventricle systolic, diastolic and global functions of young purebred Arabian horses obtained at rest in the pre- (PreTr) and post-conditioning (PostTr) periods.

SYSTOLIC FUNCTION							
	PreTr			PostTr			
	Mean ± sd	1st quartile	3rd quartile	Mean ± sd	1st quartile	3rd quartile	p
ESV (cm³)	188.1 ± 30.1	172.5	201.4	169.3 ± 42.4	135.5	196.2	0.162
FS (%)	37.01 ± 3.97	34.06	40.11	38.17 ± 5.55	34.01	42.98	0.545
EF (%)	62.52 ± 5.73	59.52	66.17	65.66 ± 7.1	60.53	71.74	0.305
VTI (cm)	31.32 ± 5.38	27.17	34.25	32.01 ± 4.23	30.13	34.33	0.651
AoCS (cm^2^)	14.78 ± 2.45	14.16	16.05	15.94 ± 2.8	14.46	18.86	0.337
SV (cm³)	467.4 ± 96.9	411.3	528.7	509.5 ± 108.6	461.8	577	0.277
SI	1.435 ± 0.345	1.16	1.63	1.594 ± 0.378	1.423	1.741	0.306
CO (l/min)	17.54 ± 4.02	15.27	19.71	18.04 ± 4.43	15.50	20.45	0.745
CI	3.134 ± 0.353	3.12	3.30	3.177 ± 0.202	3.123	3.253	0.770
Vmax (m/s)	0.958 ± 0.141	0.85	1.06	0.981 ± 0.128	0.916	1.07	0.536
TPP (ms)	139.4 ± 19.2	130	148	130.3 ± 17.3	120	140	0.210
ET (ms)	479.3 ± 71.2	455.6	492.7	471.9 ± 30.01	449.3	490.7	*0*.*266*
DT (ms)	336.8 ± 66.7	317.9	373.3	341.9 ± 25.7	338.7	356.7	*0*.*875*
PEP (ms)	135.3 ± 44.1	116	131	119.6 ± 22.8	106	127.67	*0*.*084*
PEP/ET	0.288 ± 0.102	0.25	0.29	0.256 ± 0.059	0.226	0.264	0.098
Vcf (cm/s)	0.791 ± 0.125	0.67	0.86	0.815 ± 0.136	0.699	0.879	0.490
S_1_ (m/s)	0.081 ± 0.013	0.078	0.087	0.077 ± 0.019	0.065	0.087	0.574
S_m_ (m/s)	0.113 ± 0.008	0.102	0.118	0.101 ± 0.008	0.097	0.104	**0.034**
*t*S_1_ (ms)	31.80 ± 9.65	26.67	34	28.83 ± 6.57	26	32	0.469
*t*S_m_ (ms)	93.89 ± 32.58	67	118	80.45 ± 31.99	60.67	81.33	0.221
IVCT (ms)	90.74 ± 14.56	85	98.67	90.31 ± 14.79	80	96	0.942
DIASTOLIC FUNCTION							
	PreTr			PostTr			
	Mean ± sd	1st quartile	3rd quartile	Mean ± sd	1st quartile	3rd quartile	p
EDV (cm³)	507.3 ± 64.5	493.5	539.9	489.1 ± 49.03	461.1	499.4	0.202
E_1_ (m/s)	0.079 ± 0.015	0.069	0.084	0.058 ± 0.013	0.05	0.07	< **0.001**
E_m_ (m/s)	0.330 ± 0.035	0.31	0.35	0.299 ± 0.040	0.288	0.33	**0.008**
*t*E_m_ (ms)	617.2 ± 36.2	601.3	642.7	625.8 ± 21.8	610.7	626.7	0.515
DTE_m_ (ms)	58.22 ± 19.62	48	62.67	83.89 ± 45.57	42.67	129.33	*0*.*107*
A_m_ (m/s)	0.098 ± 0.016	0.089	0.111	0.081 ± 0.010	0.07	0.09	**0.006**
E_m_/A_m_	3.452 ± 0.772	2.824	3.974	3.748 ± 0.741	3.585	4.151	0.196
IVRT (ms)	63.14 ± 10.73	57.33	70	54.56 ± 10.48	48	56	0.088
E (m/s)	0.718 ± 0.078	0.677	0.725	0.627 ± 0.077	0.576	0.689	**0.007**
*t*E (ms)	131.8 ± 23.9	120	139.3	128.5 ± 18.5	114	142	0.671
DTE (ms)	137.3 ± 39.8	111	152	133.8 ± 27.9	116	146	0.837
A (m/s)	0.383 ± 0.037	0.369	0.408	0.378 ± 0.04	0.353	0.4	0.641
E/A	1.897 ± 0.308	1.73	1.91	1.697 ± 0.231	1.55	1.83	**0.037**
E/E_m_	2.191 ± 0.267	1.997	2.4	2.167 ± 0.520	1.94	2.35	*0*.*622*
GLOBAL FUNCTION							
	PreTr			PostTr			
	Mean ± sd	1st quartile	3rd quartile	Mean ± SD	1st quartile	3rd quartile	p
MPI	0.349 ± 0.072	0.331	0.342	0.313 ± 0.0534	0.273	0.333	0.187

A_m_: LV late-diastolic radial myocardial velocity; A: left ventricle (LV) peak velocity in the late filling phase; CO: cardiac output; E1: LV peak velocity during isovolumic relaxation; E: LV peak velocity in the early filling phase; E_m_: LV peak radial myocardial velocity in the early filling phase; E/A: E to A ratio; E_m_/A_m_: E_m_ to A_m_ ratio; E/E_m_: E to E_m_ ratio; FS: fractional shortening; EF: ejection fraction; CI: cardiac index; MPI: myocardial performance index; SI: systolic index; SV: stroke volume; VTI: velocity time integral; PEP: pre-ejection time; PEP/ET: PEP to ET ratio; S_1_: LV peak radial myo-cardial velocity during isovolumic contraction; S_m_: LV peak radial myocardial velocity during systole; AoCS: aortic cross-sectional area; IVCT: isovolumic contraction time; DT: time between peak and end of aortic flow; DTE: deceleration time of E wave; DTE_m_: deceleration time of Em wave; *t*E: time between onset and peak velocity of E wave; ET: LV ejection time; *t*E_m_: time between onset of QRS complex and beginning of E_m_; TPP: time between onset and peak aortic flow; IVRT: isovolumic relaxation time; *t*S_1_: time between onset and peak radial wall motion velocity during isovolumic contraction; *t*S_m_: time between onset and peak velocity of S_m_; Vcf: mean velocity of circumferential fiber shortening; EDV: end-diastolic volume; EF: ejection fraction; Vmax: maximal velocity of aortic flow; ESV: end-systolic volume. P-values and p-values in italics were obtained by Student’s t-test and Wilcoxon rank sum test, respectively. P-values in bold indicate a statistical significance of 5%.

The results referring to the cardiovascular evaluation performed immediately after the ST are compiled in Table 3. Among the systolic variables, there were significant differences in the values of VTI, SV, SI, CI, ET, DT, PEP, Vcf, and PEP/ET ratio, while for the diastolic function differences were found in EDV, *t*E_m_ and EA/E_m_ ratio. It is worth mentioning that S_1_, *t*S_1_, IVCT, E_1_, IVRT and MPI obtained at rest were not obtained under physical stress. For the systolic function, S_1_ and *t*S_1_ were not measured due to the difficulty in determining the presence of the S_1_ wave in the spectrum obtained by TDI, hindering the calculation of IVCT. Similarly, during the evaluation of the diastolic function it was not possible to determine the E_1_ wave, and therefore the IVRT. Furthermore, there was a fusion of the E and A waves, which led to the joint evaluation of their related variables (EA, *t*EA, DTEA, EA/E *t*EA), making it difficult to calculate the E/A ratio. Since the IVCT and IVRT values were not obtained, the MPI was not calculated as well.

**Table 3:** Mean ± sd, first and third quartiles of the variables of the left ventricle systolic and diastolic functions of young purebred Arabian horses obtained immediately after the stress test in the pre- (PreTr) and post-conditioning (PostTr) periods.

SYSTOLIC FUNCTION							
	Pre-Tr			Post-Tr			p
	Mean ± sd	1st quartile	3rd quartile	Mean ± sd	1st quartile	3rd quartile	
ESV (cm³)	172.2 ± 19	165.9	180	187.7 ± 36.04	174.8	215.1	0.173
FS (%)	35.67 ± 2.08	34.15	37.32	36.67 ± 2.72	35	37.30	0.352
EF (%)	62.64 ± 2.60	60.87	64.28	63.91 ± 3.45	61.87	64.68	0.344
VTI (cm)	23.44 ± 6.27	18.75	26.67	28.62 ± 4.13	25.83	30.67	**0.022**
AoCS (cm^2^)	15.58 ± 3.89	15.18	17.71	16.38 ± 1.7	15.24	16.99	*0*.*966*
SV (cm³)	360.5 ± 129.3	288.1	430.7	460.7 ± 62.5	419.9	495.4	***0*.*021***
SI	1.142 ± 0.376	0.934	1.389	1.439 ± 0.211	1.31	1.6	**0.035**
CO (l/min)	38.29 ± 13.83	29.65	42.85	36.67 ± 5.91	33.12	40.23	*0*.*638*
CI	3.323 ± 0.166	3.26	3.40	3.240 ± 0.223	3.16	3.31	**0.023**
Vmax (m/s)	1.184 ± 0.204	1.03	1.36	1.126 ± 0.132	1.046	1.204	0.241
TPP (ms)	120.3 ± 11.7	112	127.5	129 ± 24.1	118.7	149.3	*0*.*262*
ET (ms)	281.3 ± 38.8	252	317.1	363.1 ± 41.9	332	389.7	**< 0.001**
DT (ms)	160.9 ± 39.9	129.5	190	234 ± 48.1	200	262.5	**< 0.001**
PEP (ms)	111.7 ± 19	94	128	89.60 ± 24.88	69.33	110.5	**0.009**
PEP/ET	0.414 ± 0.107	0.33	0.51	0.254 ± 0.082	0.185	0.312	**< 0.001**
Vcf (cm/s)	1.265 ± 0.152	1.15	1.34	1.033 ± 0.14	0.95	1.10	**< 0.001**
S_m_ (m/s)	0.107 ± 0.035	0.105	0.125	0.113 ± 0.01	0.11	0.123	*0*.*415*
*t*S_m_ (ms)	86.34 ± 26.04	75.17	96	70.67 ± 13.67	62	77.33	0.144
DIASTOLIC FUNCTION							
	PreTr			PostTr			p
	Mean ± SD	1st Quartile	3rd Quartile	Mean ± SD	1st Quartile	3rd Quartile	
EDV (cm³)	462.9 ± 40.6	426.4	489.2	518.7 ± 70.7	475.4	572.4	**0.045**
E_m_ (m/s)	0.248 ± 0.09	0.208	0.269	0.292 ± 0.049	0.257	0.32	0.143
*t*E_m_ (ms)	481.2 ± 49.1	445.7	522.7	564.2 ± 24.2	558	576	***0*.*002***
DTE_m_ (ms)	66.66 ± 36.67	37	84.5	59.64 ± 18.43	47	61.33	*0*.*594*
A_m_ (m/s)	0.101 ± 0.036	0.074	0.12	0.088 ± 0.024	0.075	0.092	0.398
E_m_/A_m_	2.907 ± 0.728	2.49	3.22	3.534 ± 1.115	2.75	4.1	0.155
EA (m/s)	0.775 ± 0.104	0.71	0.85	0.693 ± 0.127	0.603	0.78	0.135
*t*EA (ms)	93.48 ± 44.44	65	118.5	124.6 ± 26.7	110	136	**0.013**
DTEA (ms)	114.8 ± 41.3	114	130	140.4 ± 30.8	118.5	168	0.142
EA/E_m_	3.443 ± 1.017	2.98	3.9	2.520 ± 0.837	2.08	3.06	**0.048**

A_m_: LV late-diastolic radial myocardial velocity; CO: cardiac output; EA: LV peak velocity resulting from the fusion between filling phases; E_m_: LV peak radial myocardial velocity in the early filling phase; E/A: E to A ratio; E_m_/A_m_: E_m_ to A_m_ ratio; EA/E_m_: EA to E_m_ ratio; FS: fractional shortening; EF: ejection fraction; CI: cardiac index; SI: systolic index; SV: stroke volume; VTI: velocity time integral; PEP: pre-ejection time; PEP/ET: PEP to ET ratio; S_m_: LV peak radial myocardial velocity during systole; AoCS: aortic cross-sectional area; DT: time between peak and end of aortic flow; DTEA: deceleration time of EA wave; DTE_m_: deceleration time of E_m_ wave; *t*EA: time between onset and peak velocity of EA wave; ET: LV ejection time; *t*E_m_: time between onset of QRS complex and beginning of E_m_; TPP: time between onset and peak aortic flow; *t*S_m_: time between onset and peak velocity of S_m_; Vcf: mean velocity of circumferential fiber shortening; EDV: end-diastolic volume; EF: ejection fraction; Vmax: maximal velocity of aortic flow; ESV: end-systolic volume. P-values and p-values in italics were obtained by Student’s t-test and Wilcoxon signed-rank test, respectively. P-values in bold indicate a statistical significance of 5%.

**Fig 3:**
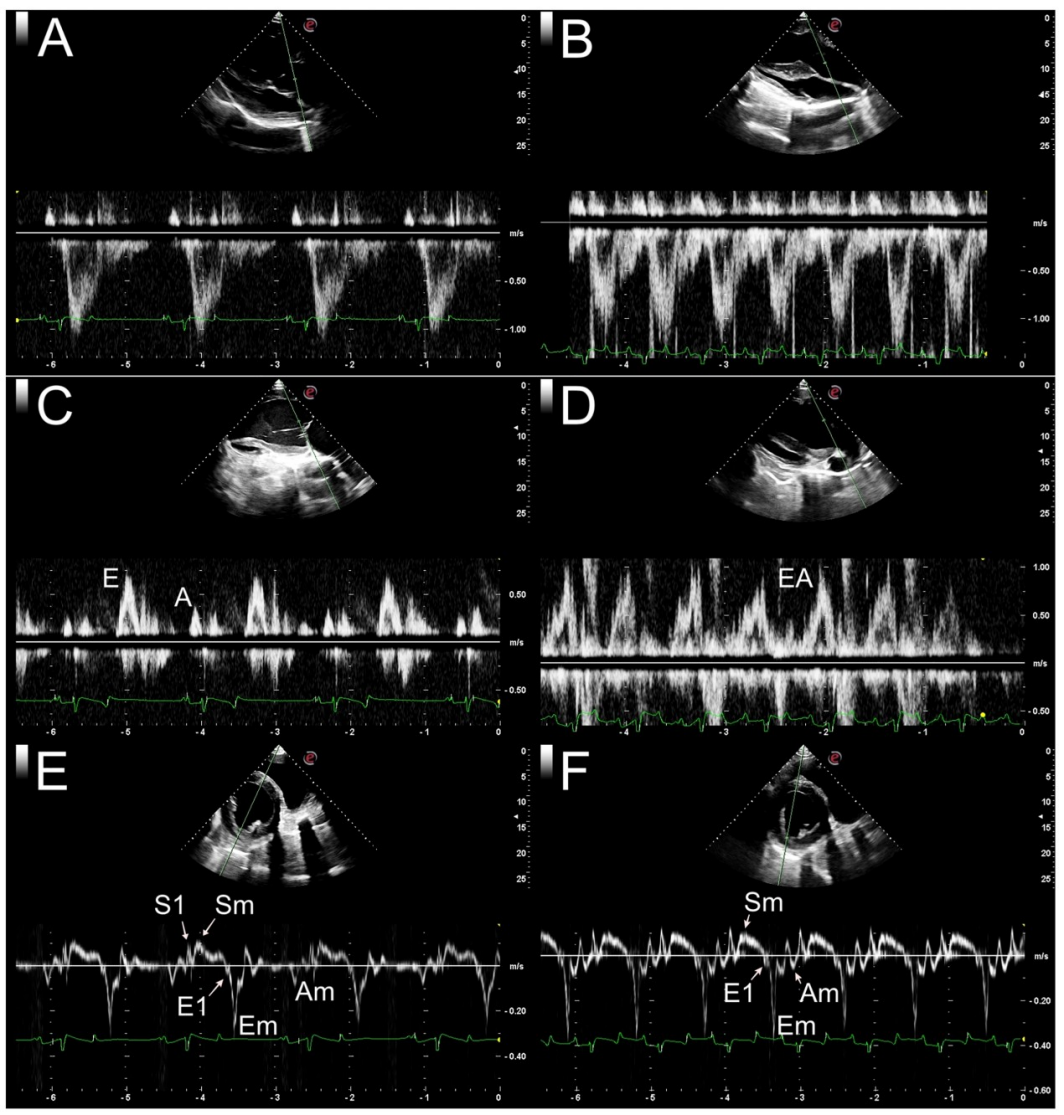
Evaluation of left ventricle (LV) systolic and diastolic parameters using pulsed-wave and tissue Doppler imaging. (A) Measurement of aortic flow at rest by the longitudinal 5-chamber image. A sample volume of 2 mm was positioned in the aortic valve opening. (B) Same image represented in A obtained immediately after the stress test. (C) Measurement of transmitral flow at rest by the longitudinal 4-chamber image. A sample volume of 2 mm was positioned in the mitral valve opening region. A: left ventricle (LV) peak velocity in the late filling phase; E: LV peak velocity in the early filling phase. (D) Same image represented in C obtained immediately after the stress test. EA: LV peak velocity resulting from the fusion between filling phases. (E) Measurement of LV radial myocardial velocity at rest obtained by the transverse image at the chordal level. A sample volume of 2 mm was positioned in the subendocardial region during diastole in order to remain in the myocardial region during systole. Am: LV late-diastolic radial myocardial velocity; E1: LV peak velocity during isovolumic relaxation; Em: LV peak radial myocardial velocity in the early filling phase; S1: LV peak radial myocardial velocity during isovolumic contraction; S_m_: LV peak radial myocardial velocity during systole. (F) Same image represented in E obtained im-mediately after the stress test. Am: LV late-diastolic radial myocardial velocity; E1: LV peak velocity during isovolumic relaxation; Em: LV peak radial myocardial velocity in the early filling phase; S_m_: LV peak radial myocardial velocity during systole.

Fig 3 illustrates the differences between the echocardiographic images obtained at rest (Fig 3A, 3C and 3E) and immediately after the ST (Fig 3B, 3D and 3F).

## Discussion

The V_2_-based conditioning program proved to be capable of promoting physiological adaptations in the animals, as well as improvements in their LV function both at rest and under physical stress. Both the V_2_ and V_4_ significantly increased by 29.8% and 31.5%, respectively. Such result indicates a greater participation of the aerobic muscle metabolism due to conditioning, which along with the increase in SV resulting from physical stress suggest a higher $\dot{\text{V}}{\text{O}}_{2}$ –calculated as the product of the HR, SV (which result in CO) and arteriovenous difference O_2_, according to Fick’s equation [45].

Another parameter that indicates improvement in the animals’ physical conditioning is the reduced HR at rest. Several studies have reported decreased HR values in human beings and rats submitted to a training program [46–49]. This response was also verified in horses [50, 51], with results similar to those observed herein. However, the mechanism responsible for such reduced HR in trained individuals has not been fully elucidated yet. Studies have shown that physical conditioning promotes intrinsic changes in the heart, especially in the sinoatrial node [47, 50]. In a study that evaluated the influence of conditioning on the intrinsic HR (determined after pharmacological blockade of the sympathetic and parasympathetic branches) of young and elderly horses, there was a decrease in this parameter in the young animals, whereas no changes were observed in the elderly animals [51]. Betros *et al*. (2013) [51] also verified a significant increase in plasma volume in young animals, which together with a reduction in intrinsic HR may explain the bradycardia promoted by conditioning, with a consequent maintenance of the ejection fraction and cardiac output, as observed in the present study. The reduction in peak HR reached immediately after physical stress observed in this study, was also reported in young human athletes, whose peak HR obtained during exercise was lower than that of control subjects [52]. When analyzed together, the reduced HR at rest and immediately after ST suggests an enhancement of HR reserve due to conditioning–an important factor for improved aerobic capacity. Nevertheless, it is not possible to state that there was an increase in HR reserve in the present study, since both the IET and the ST are submaximal intensity tests, making it difficult for the animals to reach peak HR, and consequently hindering the calculation of HR reserve.

The stress test applied was capable of significantly increasing the HR in relation to the values obtained at rest. With respect to the pre-conditioned animals, the highest HR value reached by the conditioned animals after ST was low. Such results are similar to those found in a study that investigated the influence of a six-week training program on the peak HR achieved by horses during the conditioning sessions, in which a progressive but non-significant reduction was observed [53]. The difference between the training protocol applied herein and that used by Mlyneková *et al*. (2016) [53] without association with a specific ST may explain the distinct results. Another point to consider about the highest HR value reached by the conditioned animals is that it was lower than the minimum value of 100 bpm/minutes established by the literature for echocardiographic evaluation after physical exercise [21].

There was a significant growth of animals within the experimental period. As reported, several factors can influence the development of foals, such as breed, age, sex, nutritional level, and so on [54]. In general, horses have a similar growth pattern in the period from 0 to 18 months of age, regardless of the breed and environmental conditions [55]. For PBA animals aged two years, it was observed that the TC of males and females was equivalent to 97% and 91%, respectively, of that observed in adult animals older than 60 months [56]. Similarly, the BW of two-year-old horses approaches the values observed in adult animals, as shown by Huntington *et al*. (2020) [55] for Thoroughbred foals. Despite the small differences between two-year-old horses and adult animals with regard to body development based on the above-mentioned measurements, little is known about their cardiac development during growth. Therefore, the indexing (or normalization) of certain echocardiographic variables was necessary in order to attenuate possible differences related to the animals’ development. In horses, among the proposed methods for normalizing LV dimensional measurements obtained in M-mode, those performed by exponential regression using TC are considered more accurate [57].

The concentric hypertrophic observed from the LV linear measurements obtained at rest, it was possible to observe an increase in the normalized values of IVSs and LVFWs with smaller LVIDd associated with a greater MWT and LVmass. Similar results were found in rats submitted to a six-week endurance exercise program, during which an increase in both LVmass and LVIDd in relation to the control group was observed after 12 weeks [58]. This pattern is characterized as concentric hypertrophy, a condition that is correlated with athletes who perform strength exercises classified as static or isometric and that differs from eccentric hypertrophy, a condition associated with dynamic or isotonic sports [2, 5, 6, 59]. Such patterns are also considered in studies with horses [15]. Nevertheless, a meta-analysis carried out with studies in human athletes of different modalities showed close values between the LVIDd of marathon runners and strength athletes [60]. In addition to the increase in preload mainly due to the expansion of plasmatic volume, the intense muscle mobilization observed in marathon runners also leads to a higher afterload. Likewise, endurance horses mobilize large muscle groups over long periods and are subject to significant increases in both preload and afterload. Little is known about the minimum intensity and duration of training protocols necessary to promote cardiac remodeling in non-athlete individuals with no training history [5]. Thus, when considering the conditioning period employed herein, the hypothesis is that, in the short term, young horses will present concentric adaptations. As the training period progresses, the LVIDd becomes higher, as observed by Young (1999) [15] in young 2-year-old Thoroughbred horses after 18 weeks of conditioning. The non-detection of LV volumetric alterations after short periods of training may also be related to the underestimation of both EDV and ESV by two-dimensional echocardiography [61]. More recent studies performed in humans have shown that, despite the increased LVmass, other parameters such as Vsis and FS can remain within the normal range [18].

Among the systolic variables, only S_m_ showed a reduction after conditioning, while for the diastolic function there was a reduction in E_1_, E_m_, A_m_, E and E/A ratio. The systolic function at rest is little affected in human athletes with high training loads, regardless of the type of exercise practiced [5]. In addition to the decreased HR, ventricular hypertrophy associated with a smaller LVIDd can reduce the speed of myocardial contraction, in contrast to eccentric remodeling, which causes an increase in this parameter [4]. In agreement with the present study, Rundqvist *et al*. (2016) [3] observed a reduction in the S_m_ of adolescents submitted to resistance training in relation to untrained individuals. In another study, a reduction in the global longitudinal strain was observed in Olympic athletes of different modalities when compared to control individuals [62]. These findings indicate that, as in humans, training can cause changes in the myocardial motion pattern of horses without affecting their cardiac function. However, it is important to point out that in horses only the LV circumferential velocity can be evaluated by TDI, given the difficulty in obtaining longitudinal images of the entire LV extension, which makes the determination of longitudinal velocities unfeasible. Changes in diastolic function, on the other hand, are correlated with the type of remodeling promoted by training. In humans, a decrease in diastolic parameters was reported in athletes who had concentric remodeling [5], in congruence with what was observed herein. Moreover, such alterations in diastolic function may be associated with an increase in the reserve capacity of the heart, given the greater increase in the variables when analyzed in conditioned animals after physical stress.

The echocardiographic evaluation under physical stress could detect adaptive responses in the LV function resulting from conditioning. Among the systolic parameters, CI, PEP, PEP/ET and Vcf showed a reduction under stress after conditioning. The decrease in CI is related to the equation that uses BW as a denominator and can therefore be explained by the growth of animals, together with the non-significant decrease in CO. Similarly, considering the equation used to obtain Vcf, its decrease may be associated with the increase observed in ET. Reductions in the radial wall motion of horses subjected to physical stress at a HR above 80 bpm have already been described and may be correlated with the ventricular activation pattern of mammals in general, which begins in the apical septal region with subsequent propagation to the base of the heart [63]. The reduced PEP can be explained by the greater end-diastolic pressure [64] that occurs during exercise due to higher preloads [65], which in conjunction with the increase in ET, also promotes a decrease in the PEP/ET ratio.

Heart size has an important correlation with the performance of young horses in races, especially the diastolic function variables [16]. The diastolic function is directly influenced by exercise, considering the enhanced plasma volume observed in athletes. In the long term, variations in pre- and afterload that occur during exercise sessions, among other factors, result in cardiac remodeling. In the present study, the larger EDV observed in animals conditioned after physical stress is in agreement with the results obtained in human athletes [66]. The LV filling in athletes is related to an increase in the early diastolic phase with a smaller contribution of the left atrial systole, which consequently results in a higher E/A ratio [67]. Nonetheless, it was not possible to calculate the E/A ratio under physical stress due to the fusion of waves, whose joint evaluation may explain the non-significant reduction observed herein. Another point that reflects an improvement in the diastolic function is the decreased E/E_m_ ratio, evaluated in the present study as EA/E_m_, and its correlation with LV filling pressure [68]. Lower ventricular filling pressures are associated, among other factors, with increased complacency, along with a more efficient LV relaxation, which can therefore be interpreted as a beneficial adaptive response promoted by training. However, recent studies have shown that the E/E_m_ ratio obtained under physical or pharmacological stress is not a good indicator of LV filling pressure [69, 70]. Thus, the result regarding the observed EA/E_m_ ratio should be interpreted with caution. On the other hand, the increase in the temporal variables observed after conditioning can be explained by both the higher EDV values and the lower peak HR reached in the ST, taking into account the negative correlation that exists between HR and systolic and diastolic cardiac times. Still regarding the EDV, its elevation has a direct influence on VTI, SV, SI, ET and DT, which justifies the high values detected in the present study.

Despite innovative, the present study has several limitations. The absence of a control group casts doubts over the cardiovascular changes caused by conditioning, evidenced by the growth of animals during the experimental period, with consequent structural and functional changes in their heart. Additionally, when housed in paddocks, horses can practice spontaneous physical activity, which, associated with the conditioning program, can influence the observed physiological adaptations. Another important limitation is the anatomical barriers inherent to the echocardiographic examination of horses, especially under stress, which require the operator’s movement during its execution, and that together with the high RR of the animals after exercise, can compromise the quality of the images acquired. Furthermore, during echocardiographic window change, there is a decrease in HR that can undermine the results obtained. It is also important to consider that the cardiovascular evaluation by TDI must be carefully interpreted in horses, since the myocardial velocities obtained refer only to the LV radial wall motion and contrast with studies in humans, which calculate the mean septal and lateral myocardial velocities from longitudinal images and allow the evaluation of other motion patterns.

## Conclusions

The training protocol applied promoted an increase in the aerobic capacity of young horses associated with an improvement in their LV systolic and diastolic functions both at rest and after physical stress. Stress echocardiography proved to be capable of elucidating changes in the left ventricular function not detected at rest.

## Supporting information

S1 Table Stress test protocol. (DOCX)

S2 Table Detailed conditioning protocol according to the sessions performed. V2: speed at which the lactate concentration reaches 2 mmol/l.
(DOCX)

S3 Table Variables analyzed by M-mode, pulsed-wave Doppler and tissue Doppler imaging to evaluate left ventricular function of Pure Breed Arabian horses. * The beginning or end of the waves was considered the point where the spectrum formed by pulsed-wave and tissue Doppler crossed the baseline. In cases where the tracing did not return to the baseline, the closest point to the horizontal line between two consecutive waves was used as a reference to determine the beginning or end. After stress test, in cases where there was fusion between consecutive waves, these were analyzed together.
(DOCX)
