# Supplementary material for: Effects of conditioning on the left ventricular function of young purebred Arabian horses
Source: PLoS One. 2024 Jun 3;19(6):e0304724. doi: 10.1371/journal.pone.0304724 (PMC11146711; doi:10.1371/journal.pone.0304724)
Supplement: S1 Table — (DOCX) [file pone.0304724.s001.docx]

|  | **Speed** | **Duration** | **Inclination** | **Moments** | **Determinations** |
| --- | --- | --- | --- | --- | --- |
| **Rest** | 0 m/s | - | - | T0 | Echocardiography |
| **Warm-up** | 1.7 m/s | 10 min | 0% | T1 | - |
|  | 3.2 m/s | 10 min | 0% |  |  |
| **Exercise** | 5.8 m/s | 30 min | 0% | T2 | - |
| **1º Sprint** | 7.5 m/s | 3 min | 0% | T3 | - |
| **Maintenance** | 3.0 a 3.5 m/s | 5 min | 0% | T4 | - |
| **2º Sprint** | 8.5 m/s | 2,5 min | 0% | T5 | Echocardiography |
| **Cool-down** | 1.7 m/s | 10 min | 0% | T6 | - |
