# Supplementary material for: Effects of conditioning on the left ventricular function of young purebred Arabian horses
Source: PLoS One. 2024 Jun 3;19(6):e0304724. doi: 10.1371/journal.pone.0304724 (PMC11146711; doi:10.1371/journal.pone.0304724)
Supplement: S2 Table — V2: speed at which the lactate concentration reaches 2 mmol/l. (DOCX) [file pone.0304724.s002.docx]

| **Phases** | **Duration (min)** |  | **1ª to 3ª Sessions** | |  | **4ª to 6ª Sessions** | |  | **7ª to 9ª Sessions** | |  | **10ª to 15ª Sessions** | |
| --- | --- | --- | --- | --- | --- | --- | --- | --- | --- | --- | --- | --- | --- |
|  |  |  | **Speed**  **(m/s)** | **Inclination (%)** |  | **Speed**  **(m/s)** | **Inclination (%)** |  | **Speed**  **(m/s)** | **Inclination (%)** |  | **Speed**  **(m/s)** | **Inclination (%)** |
|  |  |  |  |  |  |  |  |  |  |  |  |  |  |
| Warm-up | 05 |  | 1.6 | 0 |  | 1.6 | 0 |  | 1.6 | 0 |  | 1.6 | 0 |
|  | 05 |  | 3.4 | 0 |  | 3.4 | 0 |  | 3.4 | 0 |  | 3.4 | 0 |
|  |  |  |  |  |  |  |  |  |  |  |  |  |  |
| Training | 12 |  | 80% V_2_ | 0 |  | 80% V_2_ | 3 |  | 100% V_2_ | 3 |  | 100% V_2_ | 5 |
|  | 02 |  | Gait change | 0 |  | Gait change | 0 |  | Gait change | 0 |  | Gait change | 0 |
|  | 12 |  | 80% V_2_ | 0 |  | 80% V_2_ | 3 |  | 100% V_2_ | 3 |  | 100% V_2_ | 5 |
|  | 02 |  | Gait change | 0 |  | Gait change | 0 |  | Gait change | 0 |  | Gait change | 0 |
|  | 12 |  | 80% V_2_ | 0 |  | 80% V_2_ | 3 |  | 100% V_2_ | 3 |  | 100% V_2_ | 5 |
|  |  |  |  |  |  |  |  |  |  |  |  |  |  |
| Cool-down | 05 |  | 1.6 | 0 |  | 1.6 | 0 |  | 1.6 | 0 |  | 1.6 | 0 |
