# Supplementary material for: Effects of conditioning on the left ventricular function of young purebred Arabian horses
Source: PLoS One. 2024 Jun 3;19(6):e0304724. doi: 10.1371/journal.pone.0304724 (PMC11146711; doi:10.1371/journal.pone.0304724)
Supplement: S3 Table — * The beginning or end of the waves was considered the point where the spectrum formed by pulsed-wave and tissue Doppler crossed the baseline. In cases where the tracing did not return to the baseline, the closest point to the horizontal line between two consecutive waves was used as a reference to determine the beginning or end. After stress test, in cases where there was fusion between consecutive waves, these were analyzed together. (DOCX) [file pone.0304724.s003.docx]

| **Left ventricular systolic function** | | | |  |  | | |
| --- | --- | --- | --- | --- | --- | --- | --- |
| ***M-mode*** | | | | | | | |
| FS | Fractional shortening | $FE=\left( \frac{LVIDd-LVIDs}{LVIDd} \right)*100$ | | | | | |
| ESV | End-systolic volume | $ESV={(7*\left[ LVIDs \right]^{3})}/{(2.4+LVIDs)}$ | | | | | |
| EF | Ejection fraction | $EF=\left( \frac{EDV-ESV}{EDV} \right)*100$ | | | | | |
| EMP | Mean left ventricular free wall thickness | $EMP={(LVFWd+IVSd)}/2$ | | | | | |
| ERP | Relative left ventricular free wall thickness | $ERP={(LVFWd+IVSd)}/{LVFWd}$ | | | | | |
| LVmass | Left ventricular mass | $LVmass=1.04*(\left[ {LVIDd+LVFWd+IVSd]}^{3}- {LVIDd}^{3} \right)-13.6$ | | | | | |
| ***Pulsed-wave Doppler*** | | | | | | | |
| VTI | Velocity time integral of aortic flow | | | | $VTI=\sum V_{i}*\Delta T$ | | |
| AoCS | Aortic cross-sectional area | | | | $AoCS=*{(Ao*0.5)}^{2}$ | | |
| SV | Stroke volume | | | | $SV=VTI*CSA$ | | |
| SI | Stroke index | | | | $SI={SV}/{BW}$ | | |
| CO | Cardiac outpout | | | | $CO={(SV*HR)}/{1000}$ | | |
| CI | Cardiac index | | | | $CI={(CO+1000)}/{BW}$ | | |
| Vmax | Maximal velocity of aortic flow | | | |  | | |
| TPP | Time to peak | | | | Time between onset and peak aortic flow * | | |
| ET | Ejection time | | | | Total duration of aortic flow | | |
| DT | Deceleration time | | | | Time between peak and end of aortic flow * | | |
| PEP | Left ventricular pre-ejection period | | | | Time between onset of the QRS complex and the beginning of the aortic flow | | |
| PEP/ET | PEP to ET ratio | | | |  | | |
| Vcf | Mean velocity of circumferential fiber shortening | | | | $VCF=\frac{LVIDd-LVIDs}{\frac{LVID*ET}{1000}}$ | | |
| ***Tissue Doppler imaging*** | | | | | | | |
| S_1_ | Isovolumic contraction peak velocity | | | | Positive peak of wall motion velocity during the isovolumic contraction phase | | |
| S_m_ | Systolic radial wall motion velocity during ejection | | | | Positive peak of wall motion velocity during ejection | | |
| *t*S_1_ | Time to peak of the isovolumic contraction velocity | | | | Time between onset and peak velocity of radial wall motion during isovolumic contraciton * | | |
| *t*S_m_ | Time to peak to systolic radial wall motion velocity during ejection | | | | Time between onset and peak velocity of radial wall motion during ejection * | | |
| IVCT | Isovolumic contraction time | | | | Time between onset of S_1_ and onset of S_m_* | | |
|  |  | | | |  | | |
| **Left ventricular diastolic function** | | |  | |  | | |
| ***M-mode*** |  | | | |  | | |
| EDV | End-diastolic volume | | | | $VDF={(7*\left[ DIVEd \right]^{3})}/{(2.4+DIVEd)}$ | | |
| ***Pulsed-wave Doppler*** | | | | | | | |
| E | Early-diastolic transmitral flow velocity | | | | First positive peak velocity at the beginning of ventricular diastole | | |
| *t*E | Time to peak of E wave | | | | Time between onset and maximal early-diastolic transmitral flow velocity * | | |
| DTE | Deceleration time of E wave | | | | Time between peak and end of early-diastolic transmitral flow * | | |
| A | Late-diastolic transmitral flow velocity | | | | Second positive peak velocity at the end of ventricular diastole | | |
| E/A | E wave to A wave ratio | | | |  | | |
| ***Tissue Doppler imaging*** | | | | | | | |
| E_1_ | Isovolumic relaxation velocity | | | | Negative peak of wall motion velocity during isovolumic relaxation phase | | |
| E_m_ | Early-diastolic radial wall motion velocity | | | | Negative peak wall motion velocity during early-diastole | | |
| *t*E_m_ | Time to peak E_m_ wave | | | | Time between onset of QRS complex and beginning of E_m_ * | | |
| TDE_m_ | Deceleration time of E_m_ wave | | | | Time between peak velocity and end of E_m_ * | | |
| A_m_ | Late-diastolic radial wall motion velocity | | | | Negative peak wall motion velocity during late-diastole | | |
| E_m/_A_m_ | E_m_ to A_m_ ratio | | | |  | | |
| E/E_m_ | E to E_m_ ratio | | | |  | | |
| TRIV | Isovolumic relaxation time | | | | Time between onset of E_1_ and onset of E_m_* | | |
| **Global left ventricular function** | | | | | |  |  |
| IPM | Index of myocardial performance (Tei index) | | | | $IPM=\frac{TCIV+TRIV}{TE}$ | | |
